# Case of Retroversion of the Uterus in the Sixth Month of Pregnancy—Forcible Reduction—Peritonitis—Recovery

**Published:** 1875-10

**Authors:** Patrick Booth

**Affiliations:** Wake County, N. C.


### CASE OF RETROVERSION OF THE UTERUS IN THE SIXTH MONTH OF PREGNANCY—FORCIBLE REDUCTION—PERITONITIS —RECOVERY.

BY PATRICK BOOTH, M. D., OF WAKE COUNTY, N. C.

On the night of June 18, I was summoned in great haste to Mrs. A., a housewife; aged 26; in her third pregnancy. She complained of intense pain throughout the pelvic regions, back and thighs; had suffered with some periodical pains "since directly after Christmas;" also with constipation, but still more with retention of urine; but after a "hard day's scouring yesterday she had been worse than ever, and had suffered double death."

Upon inquiry, I found that she was not quite six months pregnant—lacking some eight or ten days. On an examination, per vaginam, I found the fundus of the uterus in the hollow of the sacrum, and the os jammed, as it were, behind the symphysis, and not dilated. The parts were of natural temperature and not congested.

After using the catheter she was placed upon her knees with her face upon the bed, with the view of attempting reduction, but the situation seemed to increase the difficulty: the fundus fell forward, the os could not be found, and the uterus *seemed* completely inverted. The difficulty of finding the neck and the os was not so much in *their* changing position, I think, as from the difficulty of raising the fundus and searching for them. I then placed her upon the side, in the "semi-prone half lateral position" and upon the back successively, and attemped reduction, fruitlessly, by vagina, by rectum, by vagina and rectum, again and again. Finally, "perplexed, but not in despair," I gave sulph. magnesia, and left my patient to find temporary relief from chloral and morphine, cautioning her to remain in the "semi-prone half lateral position," with the pelvis much elevated. I hoped that position, quietude and the purgative might assist, while I sought aid from other sources.

Ere the time for my return, I was again called and informed that Mrs. A. had suffered nearly ever since I left her. I found the os uteri somewhat dilated, and the temperature greater than when I left. She was completely frantic with pain, and uncontrollable, except for a few moments at a time. I immediately administered chloroform and sent for assistance, and in the meantime again attempted reduction by introducing my finger into the os uteri and drawing it downward while the fundus was raised, but fruitlessly.

Dr. Penny, who had been called, at last arrived, when the chloroform was "pushed," with the object of again attempting reduction, but the pulse failed, so it was withheld. After half an hour we gave chloral and morphine, which gave a refreshing sleep of an hour and a half.

The pulse being now stronger and the patient refreshed, we determined to try the chloroform again at 9 A. M., 20th, and if other means failed, the question arose: should we puncture the uterus as advised by the authorities, or introduce the hand into the vagina and, by force, carry the fundus upwards?

If the puncture should be made and the water drawn, difficulty might arise still from adhesions, if any, and public sentiment would attribute all mishaps to that; nor would we have the waters to dilate the os in the labor which was inevitable, which would render the case tedious. On the other hand, public sentiment would not condemn the use of the hand; and if that failed, we could still resort to the trocar and canuea. Provided the womb was too large to pass the promontory of the sacrum, we would have the waters to expedite the labor, and as the patient was weak and growing weaker, the quickest was the mode to be preferred; therefore we determined upon the latter, although neither of us had ever seen such procedure advised or recorded.

The chloroform was borne well, and the hand was introduced as proposed, and, with considerable force, the womb was replaced. She seemed much relieved and, under the effects of opiates, rested well, taking some nourishment, till 5 P. M., when vomiting occurred and labor-pains began, which I did not interfere with, but rather encouraged, until six hours had elapsed; then observing the exhaustion, I gave $\frac{1}{4}$ gr. of morphine with 30 grs. chloral, which produced refreshing sleep for one and one-half hours, when she awoke, and, with a single tremendous pain, the waters broke and the child was expelled (feet presenting), except the head, and arm which was up over the head. These were delivered in the usual manner, and, as the head escaped, she sank as if lifeless. Hemorrhage, thought I, and introduced my hand into the womb to find, however, no hemorrhage, but an inert uterus, excited not even by the presence of my hand. I immediately gave ergot and brandy, which produced the happiest effect. The placenta being delivered, my patient slept six or eight hours.

The next morning there was some fever and considerable soreness. I gave ext. hyoscyamus and camphor. On my return in the evening, I found considerable fever, high pulse, and soreness and pain over nearly the whole abdomen, with some tympanites in the left halves of the hypogastric and umbilical regions. A turpentine steepe was immediately applied, but produced such constitutional disturbance that it was withdrawn. The hyoscyamus was stopped and 1 gr. opium given every hour, and as this is one of the diseases that tends to death by esthenia, why not employ tonics as in pneumonia and other inflammations? Therefore I gave, also, $\frac{1}{2}$ gr. quinine with the opium, and ordered brandy occasionally. Warm poultices proved grateful to the patient and were used freely.

This treatment, with an occasional ænema (when urgent only) of soap, turpentine and castor oil, was continued for eight days, when the symptoms began to subside. Upon the eleventh day the treatment was discontinued and a purely tonic substituted. At the end of one month only the ænemic condition and enlarged spleen remained as relics, and they gradually yielded to iron and iodine.

